# Impact of secondhand smoke on air quality in partially enclosed outdoor hospitality venues: a review

**DOI:** 10.1186/s12889-024-19394-w

**Published:** 2024-07-14

**Authors:** Michael Tong, Nigel Goodman, Sotiris Vardoulakis

**Affiliations:** 1 National Centre for Epidemiology and Population Health, The Australian National University, Canberra, ACT 2601 Australia; 2 Healthy Environments And Lives (HEAL) National Research Network, Canberra, Australia

**Keywords:** Secondhand smoke, Tobacco, Air pollution, PM_2.5_, Hospitality venue, Public health

## Abstract

**Background:**

Smoking is a leading cause of premature mortality and morbidity globally. The pollutants generated from smoke are not only harmful to smokers, but also to those exposed to secondhand smoke. As a result of increasingly restrictive indoor smoke-free policies in many countries, there is a tendency for tobacco smoking to move outdoors into partially enclosed settings in hospitality venues. The aim of this systematic review was to evaluate the impact of secondhand smoke on air quality in outdoor hospitality venues.

**Methods:**

Two electronic databases PubMed and Scopus were searched from 1 January 2010 to 30 June 2022 for studies of air quality impacts from tobacco smoking in outdoor hospitality venues. A total of 625 studies were screened and 13 studies were included in this review.

**Results:**

The majority (9 studies) of reviewed studies monitored PM_2.5_ concentration as an indicator of secondhand smoke. PM_2.5_ was reported from 10.9 µg/m^3^ to 91.0 µg/m^3^ in outdoor smoking areas, compared to 4.0 µg/m^3^ to 20.4 µg/m^3^ in outdoor control sites unaffected by smoking. Secondhand smoke can also drift into adjacent outdoor areas or infiltrate into indoor environments thus affecting air quality in spaces where smoking is not permitted.

**Conclusions:**

The reviewed studies indicated that air quality within outdoor hospitality venues where smoking is permitted is unlikely to meet current World Health Organization (WHO) ambient air quality guidelines for PM_2.5_. Customers and staff in outdoor hospitality venues with active smoking, and in adjacent outdoor and indoor non-smoking areas, are potentially exposed to secondhand smoke at levels exceeding WHO guidelines. Stronger smoking control policies are recommended for outdoor hospitality venues to protect the health of customers and staff from harmful secondhand smoke exposure.

**Prospero registration:**

CRD42022342417.

**Supplementary Information:**

The online version contains supplementary material available at 10.1186/s12889-024-19394-w.

## Background

Smoking is a leading cause of premature mortality and morbidity globally [1]. The health impact of smoke is not only harmful to active smokers, but also to those who are exposed to secondhand smoke [2]. Globally, there are 1.3 billion tobacco users, which results in more than 8 million deaths as well as more than 1,800 billion (purchasing power parity) dollars of healthcare costs and productivity losses annually [3, 4]. Of those, it is estimated that more than 7 million deaths are attributed to direct tobacco smoking, and 1.2 million to secondhand smoke exposure. To a specific country for example in Australia, each year there are about 20,500 deaths attributed to tobacco smoking, which accounts for 8.6% of the national total burden of disease [5]. For other countries such as the United States, Europe, and China, 12.5%, 18.4% and 26.6% of adults are daily smokers, respectively [6–8], and more than 10% of all-cause deaths are attributed to smoking [9]. It is imperative to review the impact of secondhand smoke on air quality for further informing future smoking control policies.

It is worth noting the global prevalence of tobacco smoking is falling, while the use of e-cigarettes and shisha (i.e. an oriental tobacco pipe) is increasing globally, particularly among young people [9–11]. The lifetime prevalence of e-cigarettes globally was 23%, and even higher prevalence (25%) of e-cigarettes among adolescents [10]. Globally the prevalence of shisha ranged from 9 to 15%, surpassing the prevalence of tobacco smoking, and particularly higher among adolescents (up to 35%) [11]. E-cigarettes also represent a serious public health risk for non-smokers, which can cause a series of respiratory and cardiovascular diseases [12]. Further for dual users (e.g. smokers and vapers), there is strong evidence of increased health risks such as higher blood pressure, endothelial dysfunction, and acute effects on lung function [13]. Shisha smoke also represents a public health problem [11]. Many shisha smokers have a misconception that shisha smoking is less harmful compared with cigarette smoking [11].

Cigarette smoke is a complex mixture of air pollutants that are hazardous to health, including nicotine, carbon monoxide, benzene, formaldehyde, fine particulate matter (PM_2.5_), hydrogen cyanide, and heavy metals such as arsenic and lead [14]. Many of the compounds in tobacco smoke are also found in the emissions from e-cigarettes and other forms of smoking (e.g., shisha) [15, 16]. E-cigarette liquids can contain a wide range of hazardous compounds, including benzene, xylene, toluene, formaldehyde and acetaldehyde [17]. Major air pollutants from e-cigarette vapour include PM_2.5_, nicotine, volatile organic compounds, and heavy metals [15]. Shisha smoke is also a complex mix of chemicals, including nicotine, carbon monoxide, polycyclic aromatic hydrocarbons, aldehydes, nitric oxide, furans, and nanoparticles [16]. These hazardous pollutants, many of them carcinogenic (e.g. benzene, formaldehyde), are present in the stream of smoke or vapour inhaled by the person using the tobacco product or vaping device and by exposed bystanders.

Smoke-free policies are one of the most effective measures for restricting smoking and reducing exposure to secondhand smoke [18–21]. Smoke-free policies have developed rapidly over the last few decades internationally [22]. However, compared with widely implemented comprehensive indoor smoke-free policies, outdoor smoking control policies are less widely adopted. Due to increasingly restrictive indoor smoke-free policies, there is a tendency for tobacco smoking to move outdoors in hospitality venues, particularly in partially enclosed outdoor settings, such as designated outdoor smoking areas with or without overhead cover and partial walls in pubs, bars and restaurants, potentially affecting air quality, and personal exposure to a wide range of hazardous chemicals including particulate matter, nicotine and carbon monoxide [23–25].

In 2013, World Health Organization (WHO) revised Article 8 of the Framework Convention on Tobacco Control and recommended that outdoor or quasi-outdoor public places should be free from exposure to secondhand tobacco smoke [26]. Since then, there has been development in outdoor smoke-free policies and regulations internationally [27]. These policies and regulations mostly apply to public spaces, such as bus stations, playgrounds, schools, educational campuses, public offices, as well as hospitality venues including certain partially enclosed outdoor dining and drinking areas [28, 29].

The levels of secondhand smoke in partially enclosed outdoor hospitality venues are influenced by several factors, including the number of active smokers, the degree of enclosure (including roof, screens and walls), the weather conditions, and any operating ventilation systems [30, 31]. Further another major factor that influences the level of secondhand smoke in these outdoor hospitality venues is the effective enforcement of smoke-free policies (both indoor and outdoor) [32, 33]. Air quality in such outdoor environments, as affected by tobacco smoking, has been studied in a variety of settings in recent years [30, 31].

The aim of this systematic review is to evaluate and synthesise the scientific literature regarding the impact of secondhand smoke on air quality in partially enclosed outdoor hospitality venues in different settings, and to provide an update on previous review regarding the secondhand tobacco smoke exposure in semi-open hospitality venues in 2012 [31]. This would help better understand the effectiveness of current smoking control policies in protecting the health of customers and staff in the context of the current trend for tobacco smoking to move outdoors in hospitality venues.

## Methods

### Search strategy

A search of peer-reviewed literature published between 1 January 2010 and 30 June 2022 was conducted to establish tobacco smoking in partially enclosed outdoor hospitality venues based on the Preferred Reporting Items for Systematic Reviews and Meta-Analyses guidelines (PRISMA) [34]. The date range for publications was limited from 2010 onwards to capture the latest tobacco control policies and their impact on tobacco smoking. Two electronic databases PubMed and Scopus were searched for relevant information. Manual searches from the reference lists of included evidence were also performed to maximize retrieval of additional relevant studies. The protocol for this review was registered with PROSPERO (International Prospective Register of Systematic Reviews registration, CRD42022342417).

### Study selection and criteria

The electronic databases PubMed and Scopus were used to search for the impact of secondhand smoke on air quality in partially enclosed outdoor hospitality venues with title, abstract or full text. A search strategy of random combinations of the following keywords “air quality”, “air pollution”, “particulate matter”, “fine particles”, “aerosol”, “PM10”, “PM2.5”, “ultrafine”, “nitrogen dioxide”, “carbon monoxide”, “nicotine”, “formaldehyde”, “benzene”, “cotinine”, “acrolein”, “acetaldehyde”, “ethanol”, “d-limonene”, “xylene”, “toluene”, “isoprene”, “hexaldehyde” (outcome), paired with “tobacco”, “cigarette”, “e-cigarette”, “vapor”, “vapour”, “vape”, “vaping”, “narghile”, “shisha”, “hookah”, “pipe”, “smoke”, “secondhand” (exposure), in “partially”, “not fully”, “not completely”, “not entirely”, “semi”, “unenclosed”, “closed”, “enclosed”, “covered”, “undercover”, “confined”, “outdoor”, “designated”, “public”, “shared”, “communal”, “patio”, “dining”, “diner”, “terrace”, “venue”, “entertainment”, “hospitality”, “club”, “pub”, “restaurant”, “café”, “coffee”, “cafeteria”, “casino”, “airport”, “terminal”, “station”, “hotel”, “shop”, “bar”, “area” (setting), was used in this review. The detailed search strings are provided in Supplementary Material. First, titles and abstracts were screened for relevance and shortlisted. Next, the selected articles were obtained for full-text review and evaluated to determine whether the evidence met the inclusion criteria. The references from the selected articles were then checked for additional relevant evidence.

Studies were included if they meet the inclusion criteria: (1) peer-reviewed journal articles, (2) original research focused on smoking and air quality, and (3) in partially enclosed (not fully enclosed setting) outdoor hospitality venues. The systematic review was limited to these original research papers published in the scientific literature between 2010 and 2022 in English. Other studies were excluded from this review including: (1) studies on environmental air quality that were not related to tobacco smoke, e.g. bushfire smoke, (2) studies that were conducted in non-partially enclosed outdoor hospitality venues, (3) studies that were funded fully or partially by tobacco companies, or (4) editorials, reviews, letters, commentaries, conference abstracts, posters, books, and grey literature were also excluded.

### Screening, data extraction and synthesis

**Fig. 1:**
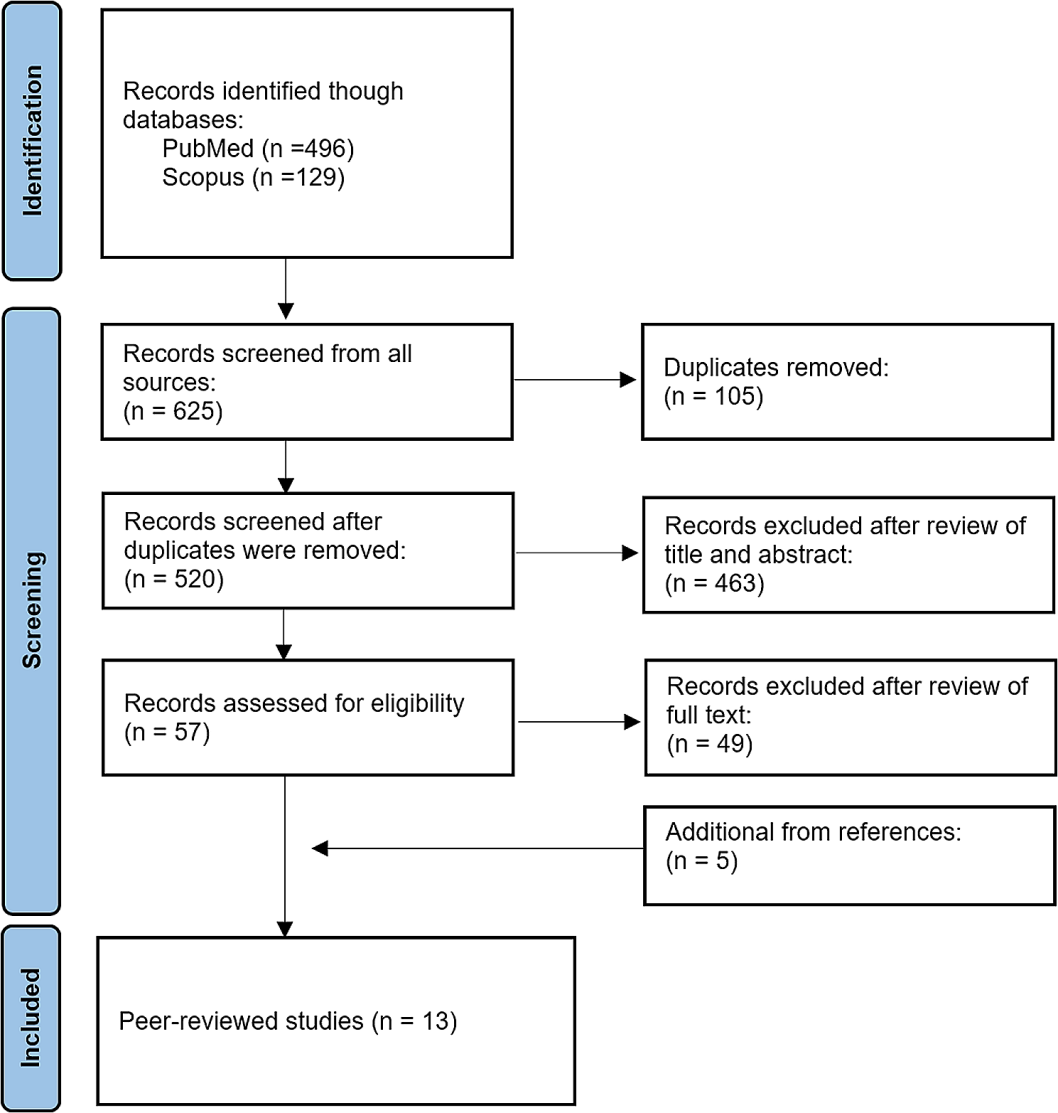
PRISMA flowchart for the literature search and selection process. The literature was included or excluded in three phases based on (a) title and/or abstract, (b) eligibility following full-text assessment, and (c) synthesis inclusion criteria. The number of records considered at each stage is indicated in brackets

All records were managed in EndNote. Duplicates were removed using the in-built software function. The screening of articles by title, abstract, and full-text selection against the eligibility criteria was undertaken independently by two reviewers. Any disagreements or inconsistencies in the screening were resolved via discussion between the reviewers. Once articles were selected for inclusion, relevant data were extracted, including author names, publication year, country/location, setting, measurement of smoke indicator (exposure) and air quality (outcome). The extraction of air quality data was categorized into three groups by measurements within outdoor smoking areas, adjacent to smoking areas, and in control sites. The study findings were summarized and synthesized narratively. Figure 1 shows the PRISMA flowchart for the literature search and selection process.

### Assessment of quality of evidence

The quality of the included studies was assessed using a modified version of the National Institutes of Health (NIH) Quality Assessment Tool for Observational Cohort and Cross-sectional Studies [35]. Studies were assessed using all the items of the NIH tool [35]. The tool evaluates the validity of a study including research question, study population, sample size, survey approaches, statistical analyses, exposures, outcomes and confounding factors, and whether quality assurance and/or quality control steps were verifiable to ensure data quality. A detailed description of the assessment is provided in Supplementary Material.

## Results

The systematic search generated 625 records that were imported into Endnote during the initial search as shown in Fig. 1. After the removal of duplicates and screening by title and abstract, there were 57 articles identified for full-text review. Of these, there were eight peer-reviewed articles plus five additional peer-reviewed articles from reference lists that met the criteria for inclusion in the review. The 13 studies included covered different geographic regions, such as Oceania, Europe and America, and specifically: Australia (*n* = 3), New Zealand (*n* = 2), Turkey (*n* = 1), Spain (*n* = 3), multiple European countries (*n* = 2), United States (*n* = 1), and Brazil (*n* = 1) (Table 1).

Of these 13 studies, 9 were of good quality and 4 were of fair quality. The “fair” quality rating was due to a relatively small number of locations or the study taking place before more comprehensive smoking restrictions were implemented. The overall level of the evidence was NIH evidence grade Good. A summary table of evidence grading is available in Supplementary Materials (Table S1).

**Table 1:** The impact of smoke on air quality in partially enclosed outdoor hospitality venues in different settings

Author, Year	Location	Settings (Sample size and Sampling time)	Secondhand smoke indicator	Monitoring within outdoor smoking area	Monitoring adjacent to smoking area	Measurement in control site
Brennan et al., 2010 [36]	Australia, Melbourne	Pubs and Bars (*n* = 19, 30 min)	PM_2.5_ (mean)	32.1 µg/m^3^ (*n* = 19, 30 min)	N/A	N/A
Cameron et al., 2010 [37]	Australia, Melbourne	Restaurants and Cafes (*n* = 69, 30 min)	PM_2.5_ (mean)	27.3 µg/m^3^ (*n* = 69, 30 min)	N/A	8.4 µg/m^3^ (*n* = 69, 5 min)
Stafford et al., 2010 [38]	Australia, Perth & Mandurah	Cafes and Pubs (*n* = 28, 20 min)	PM_2.5_ (mean)	17.0 µg/m^3^ (*n* = 28, 20 min)	N/A	4.0 µg/m^3^ (*n* = 28, 6 min)
Wilson et al., 2011 [39]	New Zealand, Wellington	Pubs, Bars and Restaurants (*n* = 20, 30 min)	PM_2.5_ (mean)	72.0 µg/m^3^ (*n* = 20, 30 min)	54.0 µg/m^3^ (*n* = 13, 25 min, indoor)	N/A
Edwards et al., 2011 [40]	New Zealand, Wellington	Pubs and Bars (*n* = 7, 30 min)	PM_2.5_ (mean)	91.0 µg/m^3^ (*n* = 6, 30 min)	71.0 µg/m^3^ (*n* = 6, 15 min, indoor)	N/A
Kaplan et al., 2019 [41]	Turkey	Restaurants, Bars, Clubs and Cafes (*n* = 72, 5 min)	PM_2.5_ (median)	31.0 µg/m^3^ (*n* = 20, 5 min)	25.0 µg/m^3^ (*n* = 72, 5 min, outdoor)	9.0 µg/m^3^ (*n* = 1, 5 min)
Sureda et al., 2018 [42]	Spain, Madrid	Pubs, Bars and Restaurants (*n* = 14, 30 min)	PM_2.5_ (median)	10.9 µg/m^3^ (*n* = 14, 30 min)	N/A	7.8 µg/m^3^ (*n* = 36, 30 min)
		Pubs, Bars and Restaurants (*n* = 14, 30 min)	Nicotine (median)	0.1 µg/m^3^ (*n* = 14, 30 min)	N/A	N/A
Fu et al., 2016 [43]	Spain, Barcelona	Restaurants and Cafes (*n* = 70, 30 min)	Nicotine (median)	0.5 µg/m^3^ (*n* = 51, 30 min)	0.4 µg/m^3^ (*n* = 49, 30 min, outdoor)	< 0.1 µg/m^3^ (*n* = 18, 30 min)
Lopez et al., 2012b [44]	Spain (multiple cities)	Restaurants, Bars and Pubs (*n* = 12, 7days)	Nicotine (median)	7.8 µg/m^3^ (*n* = 8, 7days)	1.2 µg/m^3^ (*n* = 8, 7days, outdoor)	0.1 µg/m^3^ (*n* = 8, 7days)
Lopez et al., 2012a [45]	Europe (multiple countries)	Restaurants, Bars and Clubs (*n* = 16, 30 min)	PM_2.5_ (median)	43.6 µg/m^3^ (*n* = 16, 30 min)	N/A	N/A
		Restaurants, Bars and Clubs (*n* = 20, 30 min)	Nicotine (median)	4.2 µg/m^3^ (*n* = 20, 30 min)	N/A	N/A
Henderson et al., 2021 [46]	Europe (multiple countries)	Pubs, Bars and Cafes (*n* = 21, 30 min)	Nicotine (median)	3.5 µg/m^3^ (*n* = 21, 30 min)	N/A	N/A
St Helen et al., 2011 [47]	USA, Georgia	Bars and Restaurants (*n* = 5, 15 min)	PM_2.5_ (mean)	44.6 µg/m^3^ (*n* = 3,15 min)	N/A	20.4 µg/m^3^ (*n* = 1,15 min)
		Bars and Restaurants (*n* = 5, 15 min)	CO (mean)	1.4 ppm (*n* = 3,15 min)	N/A	1.3 ppm (*n* = 1,15 min)
Issa et al., 2011 [48]	Brazil, Sao Paulo	Clubs, Bars and Restaurants (*n* = 585, N/A)	CO (mean)	3.8 ppm Restaurants (*n* = 585, N/A)	N/A	1.2 ppm Restaurants (*n* = 585, N/A)

Control site: No smokers were expected at control sites, so measurements provided information on street-level air quality in smoke-free outdoor areas. The study settings including sample size and sampling time may differ from field measurements in outdoor smoking areas, adjacent areas and control areas. “n” indicates sample size, and “min” indicates sampling time

µg/m^3^: Microgram per cubic metre

ppm: Parts per million by volume

N/A: Information not available

The majority of the studies (*n* = 9) monitored PM_2.5_ concentration as an indicator of the levels of secondhand smoke in the air [36–42, 45, 47, 49]. Of these nine studies, two studies also reported nicotine [42, 45] and one study reported carbon monoxide (CO) [47] as an additional indicator. The other three studies reported nicotine concentration [43, 44, 46], and one study only reported carbon monoxide [48] as the indicator of the levels of secondhand smoke.

Among all studies, the 30-min PM_2.5_ concentrations reported in outdoor smoking areas ranged from 10.9 µg/m^3^ to 91.0 µg/m^3^ (Table 1). The PM_2.5_ levels in indoor or outdoor places adjacent to smoking areas were up to 71 µg/m^3^, and ranged from 4.0 µg/m^3^ to 20.4 µg/m^3^ in outdoor control sites unaffected by smoking. The concentrations of nicotine ranged from 0.1 µg/m^3^ to 7.8 µg/m^3^ in outdoor smoking areas, 0.4–1.2 µg/m^3^ in adjacent to smoking areas, and 0.1 µg/m^3^ in control sites. The concentrations of CO ranged from 1.4 ppm to 3.8 ppm in outdoor smoking areas, and 1.2–1.3 ppm in control sites. An appreciation of the impact of secondhand smoke in adjacent non-smoking areas is important; however, concentrations of secondhand smoke indicators in adjacent (indoor or outdoor) areas and control sites were not reported in all studies.

A study in Melbourne, Australia reported 30-min PM_2.5_ concentration of 32.1 µg/m^3^ in 19 partially enclosed outdoor areas of pubs and bars, where smoking was permitted [36]. Another study conducted in Melbourne reported 30-min PM_2.5_ concentration of 27.3 µg/m^3^ in 69 outdoor dining areas when cigarettes were being smoked [37]. Further, a study conducted in outdoor areas of 28 cafés and pubs in Perth and Mandurah, Western Australia found that 20-min PM_2.5_ was 17.0 µg/m^3^ when two or more smokers were on site [38]. Moreover, in Wellington, New Zealand, a study conducted in seven pubs and bars reported 30-min PM_2.5_ concentration of 91.0 µg/m^3^ in partially enclosed outdoor smoking areas, and 15-min PM_2.5_ concentration of 71.0 µg/m^3^ in adjacent indoor areas [40]. The study also found that peak PM_2.5_ concentrations varied from 140 to 801 µg/m^3^ in the partially enclosed outdoor smoking areas [40]. Another study in Wellington that monitored tobacco smoke levels across 20 pubs, bars, and restaurants reported 30-min PM_2.5_ concentrations of 72.0 µg/m^3^ in outdoor smoking areas, and 25-min PM_2.5_ concentration of 54.0 µg/m^3^ in adjacent indoor areas [39].

A study conducted in Turkey across 72 hospitality venues reported 5-min PM_2.5_ concentration of 31 µg/m^3^ in partially enclosed outdoor areas where smoking was permitted, and 25 µg/m^3^ in adjacent outdoor areas [41]. In Spain, a study conducted in 14 partially enclosed outdoor hospitality venues recorded relatively low levels of PM_2.5_ (10.9 µg/m^3^) and nicotine (0.1 µg/m^3^) over 30-min sampling [42]. Two other Spanish studies reported 30-min nicotine concentrations of 0.5 µg/m^3^ and 7.8 µg/m^3^ in partially enclosed outdoor hospitality venues, 0.4 µg/m^3^ and 1.2 µg/m^3^ in adjacent outdoor areas [43, 44]. A study across eight European countries reported 30-min PM_2.5_ concentration of 43.6 µg/m^3^, and nicotine concentration of 4.2 µg/m^3^ in partially enclosed outdoor areas of hospitality venues where smoking was permitted while indoor smoking was banned [45]. Another European study reported 30-min nicotine concentration of 3.5 µg/m^3^ across 21 partially enclosed outdoor hospitality venues [46].

In the United States, a study conducted across five bars and restaurants in Georgia found 15-min PM_2.5_ concentration of 44.6 µg/m^3^ in partially enclosed outdoor spaces of these hospitality venues, which was significantly higher than PM_2.5_ levels (20.4 µg/m^3^) in control sites [47]. Further, the same study reported 15-min concentration of CO of 1.4 ppm in outdoor smoking areas and 1.3 ppm in outdoor non-smoking areas (control sites), suggesting that there was no significant difference in CO levels between these locations [47]. Lastly, a study conducted in Brazil across 585 hospitality venues reported CO concentration of 3.8 ppm in partially enclosed outdoor areas and 1.2 ppm in control sites [48].

## Discussion

According to the United Nations, air pollution is the largest environmental risk factor for human health globally [50–52]. Many air pollutants emitted from tobacco smoking can impact human health; however, those with the strongest evidence of adverse health effects are PM_2.5_, CO, nicotine and volatile organic compounds [13, 53, 54]. PM_2.5_ is of particular concern due to the increased risk of morbidity and mortality from personal exposure, even at low levels [55–57]. The WHO has revised its guidelines for a range of air pollutants, including PM_2.5_ in ambient (i.e., outdoor) air. These include a long-term (annual) exposure guideline of 5 µg/m^3^, and a short-term (24-hour) exposure guideline of 15 µg/m^3^ for PM_2.5_ [58]. The risk of adverse health effects from air pollution is significantly reduced below these levels [59], although adverse health effects may be still experienced at lower levels, particularly in sensitive individuals. This present study is an update of the previous review that evaluated the impact of secondhand smoke on air quality in outdoor hospitality venues (7 studies between 2010 and 2012) [31], with additional 6 studies included in this review.

All eligible studies included in this review found that levels of air quality, in most cases reported as PM_2.5_, within partially enclosed outdoor hospitality venues where smoking was permitted were higher than background levels observed in “control sites”. Although averaging times differed, all reviewed studies reported levels of PM_2.5_ potentially exceeding the WHO long-term (annual) exposure guideline of 5 µg/m^3^ for PM_2.5_ in partially enclosed outdoor smoking areas in hospitality venues. Specifically, 30-min PM_2.5_ concentrations ranged from 10.9 µg/m^3^ to 91.0 µg/m^3^ (Table 1). This is consistent with previous review that indicated the mean PM_2.5_ concentrations for outdoor smoking areas when smokers were present ranged from 8.32 µg/m^3^ to 124 µg/m^3^ [31]. Except for one Spanish study [42], all other reported PM_2.5_ concentrations also potentially exceeded the WHO short-term (24-hour) exposure guideline of 15 µg/m^3^ for PM_2.5_. Therefore, there is a pressing need to enforce a complete ban on smoking in these partially enclosed hospitality venues to protect both customers and staff from the detrimental effects of secondhand smoke [2, 12, 60].

Further, 30-min PM_2.5_ levels in adjacent areas of partially enclosed outdoor smoking areas ranged from 25 µg/m^3^ up to 71 µg/m^3^ (Table 1), potentially exceeding the WHO long- and short-term guidelines for PM_2.5_. It is worth noting that even with the communicating door closed almost the whole time between an outdoor smoking area and an adjacent indoor non-smoking area in pubs in Wellington, New Zealand, 30-min PM_2.5_ was 25 µg/m^3^ in the adjacent “smoke-free” indoor areas [40]. With the communicating door intermittently open PM_2.5_ in the adjacent indoor area increased to 85 µg/m^3^, and with the communicating door open all or almost all the time PM_2.5_ further increased to 117 µg/m^3^ [40]. Therefore, customers and staff were likely exposed to unhealthy secondhand smoke levels in partially enclosed outdoor hospitality venues where smoking was permitted, as well as in adjacent outdoor and indoor areas of these venues.

This review found that the greatest contributor to the levels of secondhand smoke was the number of active smokers in the space [37, 38, 41, 43, 47, 49]. For example, one study from Turkey found that 5-min PM_2.5_ concentrations from secondhand smoke in partially enclosed outdoor hospitality venues increased from 9 µg/m^3^ when no smokers were present, to 28 µg/m^3^ when 1–10 smokers were present, to 38 µg/m^3^ when 11–20 smokers, to 49 µg/m^3^ when more than 20 smokers were present [41]. A similar finding was revealed by Stafford et al. (2010) in Australia indicating 20-min PM_2.5_ concentrations in partially enclosed outdoor hospitality venues for no smokers, one smoker, two smokers, and more than two smokers were 4.0 µg/m^3^, 10.6 µg/m^3^, 14.3 µg/m^3^, and 17.0 µg/m^3^, respectively [38]. This is also consistent with a study from Spain reporting 30-min nicotine concentrations in partially enclosed outdoor hospitality venues for 1–3 smokers, 4–6 smokers, and more than 6 smokers that were 0.3 µg/m^3^, 0.5 µg/m^3^, and 1.2 µg/m^3^, respectively [43].

The degree of enclosure of an outdoor smoking environment is another factor that can potentially affect the levels of secondhand smoke [37, 38]. A study in Melbourne, Australia, found that outdoor overhead cover increased secondhand smoke (PM_2.5_) levels by 51%, compared with no overhead cover [37]. Another Australian study (in Perth and Mandurah) also indicated that PM_2.5_ concentrations increased when the overhead coverage increased in partially enclosed environments [38]. However, one study from Spain found that there was no significant difference in outdoor air quality with the degree of enclosure (including overhead cover and vertical enclosure) in relation to secondhand smoke in a partially enclosed environment [42]. A study across 11 European countries found that when there were two or fewer smokers in a partially enclosed outdoor venue, the concentration of secondhand smoke (nicotine) increased significantly with the degree of enclosure of the venue. However, when more than two smokers were present nicotine concentrations did not significantly vary across the different levels of enclosure [46].

The proximity of the measurement device to active smoking in partially enclosed outdoor smoking areas was associated with increased levels of secondhand smoke [49]. A field experiment simulating outdoor smoking facilities in Korea found that 30-min PM_2.5_ levels were relatively low (< 5 µg/m^3^) when measurements were taken 3 meters away from the entrance of a semi-closed smoking facility [49]. In another outdoor experimental study conducted on the rooftop of a building in Korea, Hwang et al. found that 13-min PM_2.5_ concentrations from secondhand smoke were 72.7 µg/m^3^ and 11.3 µg/m^3^ at 1 and 3 meters away from the source, respectively [61]. Concentrations were found to decline further from 4.1 µg/m^3^ to 2.6 µg/m^3^ at 6 and 9 m away from the smoking source [61]. These findings suggest that outdoor smoking areas with a sufficiently large buffer zone (3 meters or more) may reduce smoke drift to non-smoking areas [49]. However, these findings should be interpreted with caution as they were conducted in experimental settings not directly comparable to partially enclosed outdoor hospitality venues.

Wind conditions were reported as another factor affecting the levels of secondhand smoke (PM_2.5_) in partially enclosed outdoor settings [38, 49], with increasing wind speed decreasing the levels of secondhand smoke, and significantly higher PM_2.5_ concentrations observed downwind from the smoking source than upwind.

Internationally, regulations that specify smoke-free and designated outdoor smoking areas vary widely [25, 62–64]. Moreover, there is divergence regarding the stringent enforcement and compliance with these smoke-free regulations and policies in both indoor and outdoor public spaces [65–67]. However, the reviewed studies indicate that current smoke-free policies with partial smoking bans or legal exemptions, such as designated smoking areas in hospitality venues, did not provide adequate protection to non-smokers [43–46]. A study from Spain revealed that there was a significant improvement of air quality in the indoor environment after implementation of a partial smoking ban in bars and restaurants, while simultaneously outdoor concentration of nicotine increased by 40% (from 7 to 10 µg/m^3^) [44]. Another study in hospitality venues in 8 European countries also found that air quality declined in outdoor areas of bars and restaurants after implementation of a partial smoking ban policy [45]. This could be because of customers moving outdoors to smoke in partially enclosed areas where smoking was permitted after the implementation of the indoor smoking ban.

There is a high level of public awareness that secondhand smoke reduces outdoor air quality and can adversely affect population health in partially enclosed public spaces of hospitality venues [68–70]. This could be an important basis for extending smoke-free policies to partially enclosed outdoor areas where smoking is currently permitted. The majority of the public, including both smokers and non-smokers, supported smoke-free policies to be extended in partially enclosed public places according to surveys conducted in the United States, Canada, Australia, China, and European countries [68–75]. Moreover, the latest study also found there was substantial public support for smoke-free policies in outdoor spaces, particularly in areas frequented by children [60].

The use of e-cigarettes and shisha is increasing, particularly among younger people in Australia, Malaysia, United Kingdom, United States, and other western countries [11, 13, 76, 77]. Elevated levels of PM_2.5_ and other pollutants can be present during outdoor use of e-cigarettes. A study in an outdoor setting in California revealed that during e-cigarette use, 5-min PM_2.5_ concentrations were 50 µg/m^3^, 15 µg/m^3^ and 5 µg/m^3^ at distances of 1 m, 2 m and 3m from the source, respectively [78]. Similarly, outdoor levels of PM_2.5_ from shisha smoking can also be considerable. For instance, a cross-sectional study of outdoor Malaysian shisha smoking centres found average PM_2.5_ concentrations of 65 µg/m^3^ (range: 27–148 µg/m^3^) over a three-hour period [77]. These limited findings indicate a need for more monitoring studies to assess air quality in partially enclosed outdoor hospitality venues with e-cigarette vaping or shisha smoking.

The experimental methods used in the reviewed studies were generally of a high standard and followed established protocols. Different approaches for venue selection were reported, including cross-sectional, random selection, and convenience sampling (Supplementary Materials, Table S1). Most studies utilised quality portable instruments for particle measurements (e.g., TSI SidePak AM510), and included details of calibration procedures [36–38, 45, 47], such as zero referencing [37, 38], the application of correction factors derived from Federal Equivalent Method instruments [45], or pump calibration using a flow meter at specified intervals (i.e., when nicotine samples were collected) [46]. The main limitations of the methods reported in the studies included: a relatively low number of air quality samples collected and locations investigated; venue selection based on a convenience sample; and relatively short sampling times (which were typically 30 min) (Table 1). In each country or state, the definitions of partially enclosed venues also varied, encompassing factors such as the percentage of enclosure, the presence of a partial or full roof, all of which can influence air quality measurements in partially enclosed outdoor hospitality venues.

Shorter sampling times fail to capture the temporal variation in the levels of pollutants, and can limit comparisons to air quality guidelines. Further, differences in sampling techniques, and in particular the sampling duration need to be recognised when comparing findings among the studies and against WHO guidelines. Moreover, our review did not find any studies to inform about the level of exposure from e-cigarette and shisha use in partially enclosed outdoor hospitality venues, which implied further research on this topic. Lastly, due to the substantial heterogeneity of methods, exposure levels and limited estimates across the included studies, a meta-analysis was not performed.

## Conclusions

Air quality within partially enclosed outdoor hospitality venues where smoking is permitted potentially exceeded the WHO ambient air quality guidelines for long- or short-term exposure to PM_2.5_ in almost all the studies included in this review. Customers and staff in partially enclosed outdoor hospitality venues where smoking is permitted, and in adjacent outdoor and indoor non-smoking areas, were potentially exposed to harmful levels of air pollution. In order to minimize harmful exposure to secondhand smoke (both indoors and outdoors), it is recommended that smoke-free regulations are extended to outdoor settings, including more stringent smoke-free policies for partially enclosed outdoor spaces in hospitality venues. Regulations to restrict the degree of communication and proximity of smoking areas to non-smoking areas are likely to reduce exposure to PM_2.5_. Adopting a complete smoking ban in partially enclosed outdoor environments, and raising awareness of the health impact of smoking in public places, would minimize the exposure of customers and staff to harmful secondhand smoke. An increasing proportion of the public, including both smokers and non-smokers, is likely to support smoke-free policies to be extended in partially enclosed public places.

### Electronic supplementary material

Below is the link to the electronic supplementary material.

Supplementary Material 1

## Data Availability

All data generated or analysed during this study are included in this published article and its supplementary information files.
