# Supplementary material for: Impact of secondhand smoke on air quality in partially enclosed outdoor hospitality venues: a review
Source: BMC Public Health. 2024 Jul 14;24:1872. doi: 10.1186/s12889-024-19394-w (PMC11247721; doi:10.1186/s12889-024-19394-w)
Supplement: Supplementary file 1 — Supplementary Material 1 [file 12889_2024_19394_MOESM1_ESM.docx]

**Title:** Impact of secondhand smoke on air quality in partially enclosed outdoor hospitality venues: A review

**Supplementary materials**

| **Detailed search strings** |
| --- |
| PubMed search strings  (air quality OR pollut* OR particulate matter OR fine particles OR aerosol* OR PM10 OR PM2.5 OR ultrafine OR nitrogen dioxide OR carbon monoxide OR nicotine OR formaldehyde OR benzene OR cotinine OR acrolein OR acetaldehyde OR ethanol OR d-limonene OR xylene OR toluene OR isoprene OR hexaldehyde) **AND**  (tobacco OR cigarette OR e-cigarette OR vapor OR vapour OR vape OR vaping OR narghile OR shisha OR hookah OR pipe OR smok* OR secondhand) **AND**  (partially OR fully OR completely OR entirely OR semi) **AND**  (unenclosed OR enclosed OR covered OR undercover OR confined OR indoor OR outdoor OR designated) **AND**  (public OR shared OR communal OR patio* OR dining OR diner* OR terrace* OR venue* OR entertainment OR hospitality OR club OR clubs OR pub OR pubs OR restaurant* OR café* OR coffee OR cafeteria* OR casino* OR airport* OR terminal* OR station* OR hotel* OR shop* OR bar OR bars OR area OR areas)  Records: **496** (all fields; restricted to human and English; published since 2010) |
| Scopus search strings  (“air quality” OR pollut* OR “particulate matter” OR “fine particles” OR aerosol* OR PM10 OR PM2.5 OR ultrafine OR “nitrogen dioxide” OR “carbon monoxide” OR nicotine OR formaldehyde OR benzene OR cotinine OR acrolein OR acetaldehyde OR ethanol OR d-limonene OR xylene OR toluene OR isoprene OR hexaldehyde) **AND**  (tobacco OR cigarette OR e-cigarette OR vapor OR vapour OR vape OR vaping OR narghile OR shisha OR hookah OR pipe OR smok* OR secondhand) **AND**  (partially OR fully OR completely OR entirely OR semi) **AND**  (unenclosed OR closed OR enclosed OR covered OR undercover OR confined OR indoor OR outdoor OR designated) **AND**  (public OR shared OR communal OR patio* OR dining OR diner* OR terrace* OR venue* OR entertainment OR hospitality OR club OR clubs OR pub OR pubs OR restaurant* OR café* OR coffee OR cafeteria* OR casino* OR airport* OR terminal* OR station* OR hotel* OR shop* OR bar OR bars OR area OR areas)  Records: **129** (title and abstract; restricted to human and English; published since 2010) |

**Table S1.** Summary of evidence grading.

| **Author, Year** | **Title** | **Comments (strengths and weaknesses)** | **Quality** | **Relevance** |
| --- | --- | --- | --- | --- |
| Brennan et al., 2010 [1] | Second-hand smoke drift: Examining the influence of indoor smoking bans on indoor and outdoor air quality at pubs and bars | *Strengths:* Good methods section, included some details of calibration and comparisons of sensor data (for drift). Clear categories for the degree of enclosure of the outdoor area.  *Weaknesses:* Relatively low number of locations; Quality of outdoor air uncertain. Unclear if there were other indoor source of PM_2.5_ (e.g., cooking, candles); Season was autumn and spring. It may have been better to focus on winter summer (i.e., extremes). | Good | High |
| Cameron et al., 2010 [2] | Second-hand smoke exposure (PM_2.5_) in outdoor dining areas and its correlates | *Strengths:* Highest number of locations sampled among the included studies; Good calibration procedure, including reference to zero-calibration using a HEPA filter; Repeated measurements done; Statistical analysis was clear.  *Weakness:* Method/approach was unique / unusual (e.g., waiting for a smoker to turn up). This may make findings difficult to compare to other studies; Weather and wind direction/speed not reported; Venues were a convenience sample in a limited number (five) geographical areas across Melbourne. | Good | High |
| Edwards et al., 2011 [3] | Smoking outdoors at pubs and bars: is it a problem? An air quality study | *Strengths:* Followed methods from others (i.e., Global Air Monitoring Study) making comparability of data stronger to similar studies; Summer and winter sampling completed so different building operational conditions explored (but likely in different buildings each time); Well defined calibration procedures; Comparisons between sensors reported giving confidence in instruments; Ambient (street measurements collected before and after).  *Weaknesses:* Small number of locations sampled; No duplicate measurements reported; Low # of measurements, i.e., one to three per pub; Some variation in the positioning of the instruments between first two nights and subsequent sampling periods; While this was intentional to answer a different research question, data is very limited; Some elevated levels of ambient PM_2.5_ (range: 8-21 ug/m^3^) may have impacted results. | Fair | High |
| Kaplan et al., 2019 [4] | Evaluation of Secondhand Smoke Using PM2.5 and Observations in a Random Stratified Sample in Hospitality Venues from 12 Cities | *Strengths:* The study used a random strategy to sample hospitality venues including indoor, outdoor and patio, which is a major strength as most previous studies assessing PM2.5 concentrations in hospitality venues, in Turkey and in other countries, have relied on convenience sampling.  *Weaknesses:* Measured PM2.5 in the outdoor areas of venues is not a specific marker for tobacco smoking in outdoor areas and could dissipate quickly. The measurement could be affected by other sources of PM2.5 outdoors. PM2.5 was measured for five minutes outdoors; five minutes might be too short a time to capture the real PM2.5 concentration caused by smoking, which was related to the challenge of conducting such a comprehensive  observational study and fieldwork in such a large number of venues. It is unable to determine if our results are representative of other small cities, towns, and communities in Turkey or whether compliance in rural areas of Turkey is similar to that which we recorded. | Good | High |
| Lopez et al., 2012a [5] | Exposure to secondhand smoke in terraces and other outdoor areas of hospitality venues in eight European countries | *Strengths:* The study stratified the selection of the venues by main potential confounders to minimize potential selection. The study recorded two of the main factors affecting outdoor secondhand smoke exposure: the type of outdoor area (semi-closed or open) and the number of smokers.  *Weaknesses:* The study used a convenience sampling of hospitality venues, which could affect the study external validity. The study did not account for factors that may affect outdoor secondhand smoke, e.g. distance and position of smokers relative to sampling equipment and wind speed or direction. | Good | High |
| Sureda et al., 2018 [6] | Second-hand smoke exposure in outdoor hospitality venues: Smoking visibility and assessment of airborne markers | *Strengths:* The study measured secondhand smoke from both summer and fall finding that there were no differences in signs of tobacco consumption between two seasons in outdoors, as well as number of roofs and/or walls, number of people smoking and the number of lit cigarettes during the measurements.  *Weaknesses:* The study collected data on weekdays and mostly between 5pm and 9pm. These times were chosen to ensure that most hospitality venues would be open during the data collection. However, some pubs and nightclubs were closed at the time of the observation. Further, it is possible that during nights and weekends less compliance with the regulation due to less fear of inspection. Future studies should consider including measurements during weekends and at night to get a real approach of exposure to secondhand smoke and compliance with the legislation in outdoor hospitality venues. | Good | High |
| Stafford et al., 2010 [7] | Second-hand smoke in alfresco areas | *Strengths:* Clear study design and straightforward and concise statistical analysis; Calibration procedure for SidePak AM510 instruments reported and included calibration factor and zero calibration procedure; Moderately high number of locations; Attempted to control for factors such as proximity to a busy road, wind level, and degree of enclosure; Used multiple linear regression analysis to explore this; Noted that PM_2.5_ decreased in windier conditions, increased when the covering increased, and increased with the number of patrons, on busy roads and with the amount of traffic.  *Weaknesses:* Variable sampling periods reported depending on the number of active smokers, (e.g., "a minimum of 15 mins of PM_2.5_ measurements"). | Good | High |
| St Helen et al., 2011 [8] | Particulate matter (PM2.5) and carbon monoxide from secondhand smoke outside bars and restaurants in downtown Athens, Georgia | *Strengths:* Long-time real-time measurements; Detailed description of monitoring locations; Well defined calibration procedures.  *Weaknesses:*  Convenience sampling; Small number of locations sampled; Other factors e.g. cooking, wind speed, temperature and humidity that may also affect the measurements. | Fair | High |
| Wilson et al., 2011 [9] | A persisting second-hand smoke hazard in urban public places: results from fine particulate (PM_2.5_) air sampling | *Strengths:* Standard methods used, included an assessment of potentially confounding factors (although did not control for these).  *Weaknesses:* Convenience sample of a relatively small number of locations over a long period of time; Some very early data (pre 2010) from a period before more comprehensive smoking bans were in place. | Fair | High |
| Fu et al., 2016 [10] | Second-hand smoke exposure in indoor and outdoor areas of cafés and restaurants: Need for extending smoking regulation outdoors? | *Strengths:* The use of vapor-phase nicotine to investigate tobacco smoke exposure is a strength of investigation, because it is tobacco-specific and very sensitive at low concentrations.  *Weaknesses:* The best observation point was not always possible because it had to be chosen from the available free tables; Other factors e.g. wind speed and direction were not controlled. | Good | High/  Medium |
| Henderson et al., 2021 [11] | Second-hand smoke exposure assessment in outdoor hospitality venues across 11 European countries | *Strengths:* Very good methodological approach, in particular for sampling (e.g., use of Defender 510 for flow calibration of pumps); (When using precision pumps for air quality sampling regular flow calibration is important.); Used socioeconomic status (SES) to help choose venues, however, still a convenience sample; Good statistical design and execution; Noted non-normal distribution of data and implemented appropriate methods of analysis (e.g., U-Mann Whitney or Kruskal Wallis tests).  *Weaknesses:* Convenience sample of venues. Relatively limited number of locations (n=20) that were only sampled on one occasion; Sampling duration of 30 minutes (although this is common); Sampled during on weekdays, which was potentially not during busiest periods (e.g., weekends), so maybe an underestimate of levels; No weather or wind conditions reported; Statistical analysis combined data from all countries (n=220). | Good | High/  medium |
| Lopez et al., 2012b [12] | Two-year impact of the Spanish smoking law on exposure to secondhand smoke: evidence of the failure of the ‘Spanish model’ | *Strengths:* The study sampled from eight different regions of Spain, representing different district demographic, cultural and sociodecnomic context within Spain.  *Weaknesses:* Convenience sampling; Although different regions of Spain were sampled, small number of locations selected; Short duration of meansurements. | Fair | High |
| Issa et al, 2011 [13] | The effect of Sao Paulo's smoke-free legislation on carbon monoxide concentration in hospitality venues and their workers | *Strengths:* The study measured pre-ban and post-ban mean CO level according to venue type and sampled environment. Totally, there are 585 sampling hospitality venues.  *Weaknesses:* Convenience sample of venues; The samples were obtained during the busiest period of the week on Thursdays, Fridays or Saturdays between 21:00 and 3:00 without specific sampling length of time. | Good | Medium |

**Table S2.** Air quality standards - Ambient particulate concentration and carbon monoxide standards from WHO

| Pollutant | Averaging period | WHO |
| --- | --- | --- |
| PM_2.5_ | Long-term (1-year) | 5 μg/m^3^ |
| PM_2.5_ | Short-term (24-hour) | 15 μg/m^3^ |
| CO | Short-term (24-hour) | 4 mg/m^3^ |

μg/m^3^: microgram per cubic metre

mg/m^3^: milligram per cubic metre

Note: Adapted from WHO global air quality guidelines [14]

**References**

1. Brennan E, Cameron M, Warne C, Durkin S, Borland R, Travers MJ, et al. Secondhand smoke drift: examining the influence of indoor smoking bans on indoor and outdoor air quality at pubs and bars. Nicotine Tob Res. 2010;12:271-7.

2. Cameron M, Brennan E, Durkin S, Borland R, Travers MJ, Hyland A, et al. Secondhand smoke exposure (PM2.5) in outdoor dining areas and its correlates. Tob Control. 2010;19:19-23.

3. Edwards R, Wilson N. Smoking outdoors at pubs and bars: is it a problem? An air quality study. N Z Med J. 2011;124:27-37.

4. Kaplan B, Carkoglu A, Ergor G, Hayran M, Sureda X, Cohen JE, et al. Evaluation of Secondhand Smoke Using PM2.5 and Observations in a Random Stratified Sample in Hospitality Venues from 12 Cities. Int J Environ Res Public Health. 2019;16.

5. López MJ, Fernández E, Gorini G, Moshammer H, Polanska K, Clancy L, et al. Exposure to secondhand smoke in terraces and other outdoor areas of hospitality venues in eight European countries. PLoS One. 2012;7:e42130.

6. Sureda X, Bilal U, Fernández E, Valiente R, Escobar FJ, Navas-Acien A, et al. Second-hand smoke exposure in outdoor hospitality venues: Smoking visibility and assessment of airborne markers. Environmental Research. 2018;165:220-7.

7. Stafford J, Daube M, Franklin P. Second hand smoke in alfresco areas. Health Promot J Austr. 2010;21:99-105.

8. St Helen G, Hall DB, Kudon LH, Pearce J, Baptiste S, Ferguson S, et al. Particulate matter (PM2.5) and carbon monoxide from secondhand smoke outside bars and restaurants in downtown Athens, Georgia. J Environ Health. 2011;74:8-17.

9. Wilson N, Edwards R, Parry R. A persisting secondhand smoke hazard in urban public places: results from fine particulate (PM2.5) air sampling. N Z Med J. 2011;124:34-47.

10. Fu M, Fernández E, Martínez-Sánchez JM, San Emeterio N, Quirós N, Sureda X, et al. Second-hand smoke exposure in indoor and outdoor areas of cafés and restaurants: Need for extending smoking regulation outdoors? Environ Res. 2016;148:421-8.

11. Henderson E, Continente X, Fernández E, Tigova O, Cortés-Francisco N, Gallus S, et al. Secondhand smoke exposure assessment in outdoor hospitality venues across 11 European countries. Environ Res. 2021;200:111355.

12. López MJ, Nebot M, Schiaffino A, Pérez-Ríos M, Fu M, Ariza C, et al. Two-year impact of the Spanish smoking law on exposure to secondhand smoke: evidence of the failure of the 'Spanish model'. Tob Control. 2012;21:407-11.

13. Issa JS, Abe TM, Pereira AC, Megid MC, Shimabukuro CE, Valentin LS, et al. The effect of Sao Paulo's smoke-free legislation on carbon monoxide concentration in hospitality venues and their workers. Tob Control. 2011;20:156-62.

14. World Health Organization. WHO global air quality guidelines: particulate matter (PM2.5 and PM10), ozone, nitrogen dioxide, sulfur dioxide and carbon monoxide Geneva: World Health Organization; 2021. Available from: <https://apps.who.int/iris/handle/10665/345329>.
